# Supplementary figures and images for: The prevalence and associated factors of the minimum acceptable diet among children aged 6–23 months in Ethiopia: A community-based cross-sectional study
Source: PLoS One. 2024 Dec 27;19(12):e0315121. doi: 10.1371/journal.pone.0315121 (PMC11676783; doi:10.1371/journal.pone.0315121)

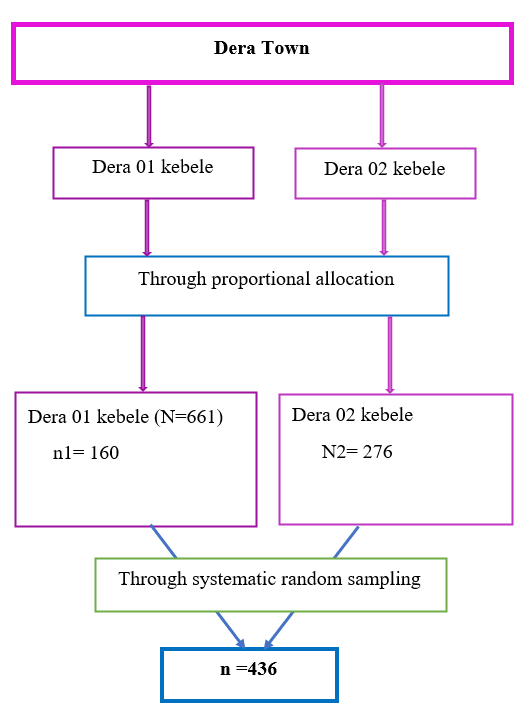

Supplement: S1 Fig — (PNG) [file pone.0315121.s001.png]
